# Photobiomodulation in Orthodontics: Mechanisms and Clinical Efficacy for Faster Tooth Movement

**DOI:** 10.7759/cureus.59061

**Published:** 2024-04-26

**Authors:** Afnan M Alzahrani, Faisal J Aljibrin, Abdulrahman M Alqahtani, Rawan Saklou, Ismail A Alhassan, Abdullah H Alamer, Mohammed H Al Ameer, Manar S Hatami, Feras Y Dahhas

**Affiliations:** 1 General Dentistry, Ministry of Health, Riyadh, SAU; 2 General Dentistry, Zara Dental Clinic, Riyadh, SAU; 3 Dentistry, Security Forces Hospital, Dammam, SAU; 4 Dentistry, Ministry of Health, Sakaka, SAU; 5 Dentistry, Ministry of Health, Al-Ahsa, SAU; 6 Dentistry, Ministry of Health, Al-Hofuf, SAU; 7 General Dentistry, Ministry of Health, Jeddah, SAU; 8 Dentistry, Al-Noor Specialist Hospital, Makkah, SAU

**Keywords:** laser dentistry, orthodontic movement, accelerated orthodontics, photobiomodulation therapy, orthodontic therapy

## Abstract

Accelerated orthodontics has revolutionized traditional dental practices by employing innovative techniques to expedite tooth movement and enhance treatment outcomes. Among these advancements, low-level laser therapy (LLLT) has emerged as a promising adjunctive method that offers a non-invasive and efficient approach to accelerate orthodontic tooth movement. By harnessing the power of low-level lasers, LLLT aims to stimulate cellular activity, promote bone remodeling, and reduce treatment duration, thereby revolutionizing the landscape of orthodontic care. In this review, we discuss the mechanism of action, methods, efficacy, advantages, limitations, and future scope of LLLT, uncovering its transformative impact on the field of accelerated orthodontics.

## Introduction and background

While orthodontic treatments have made remarkable progress in correcting malocclusions and improving oral health, the current methodologies have several limitations. An extended amount of time, ranging from months to several years, is needed to achieve the desired results with traditional orthodontic treatments. The complexity of malocclusion, patient compliance, and clinical expertise further influence the duration of treatment. Long treatment times can test a patient’s commitment and reduce compliance. Maintaining motivation and adherence to oral care routines and appointments over an extended period could be challenging for patients. Accelerated orthodontic techniques aim to address these disadvantages by reducing treatment duration, offering patients a faster and potentially more comfortable path to achieving their desired results with fewer associated challenges [1].

Accelerated orthodontics represents a dynamic evolution in the field of dentistry and is reshaping the traditional timelines of orthodontic treatments. This innovative approach is designed to expedite teeth movement and significantly reduce the duration of orthodontic therapy. By leveraging cutting-edge techniques and technologies, accelerated orthodontics aims to optimize the alignment of teeth while minimizing treatment duration and, therefore, provide patients with quicker, more efficient, and aesthetically pleasing outcomes [2].

At present, various pharmaceutical therapies are being utilized to accelerate orthodontic tooth movements. For instance, prostaglandins E1 and E2, ibuprofen, and acetaminophen injections promote tooth movement by influencing bone remodeling. Other modalities that have attracted scientific researchers are electromagnetic field, electric current, corticotomy, and mechanical vibration techniques. Applying electromagnetic forces to teeth through electromagnetic fields has been found to accentuate tooth movements [3]. In addition, the electric stimulation of the periodontal ligament and surrounding bone has been shown to alter their cellular mechanism and facilitate faster tooth movement [4]. To date, surgical exposure of cortical bone followed by corticotomy and piezocision are the most reliable methods of stimulating bone resorption and regeneration. However, their drawback is the intervention’s surgical nature, making it less appealing to the patient [5]. Ongoing research and innovation in orthodontics have focused on addressing these limitations and developing more efficient, comfortable, and predictable treatment modalities to enhance patient experiences and outcomes. The increasing scientific evidence supporting the use of low-level laser (LLL) is based on the premise that the present strategies utilized for accelerating orthodontic tooth movement are either invasive or have side effects.

Low-level laser therapy (LLLT) serves as an adjunctive therapy that complements traditional orthodontic methods by potentially reducing treatment times and improving overall treatment outcomes. Essentially, the orthodontic tooth movement is a biological response to external forces. These forces create a tension side and a pressure side. On the tension side, bone resorption occurs. All these things occur due to periodontal and osseous remodeling. LLLT triggers these remodeling processes in hydrophilic oral tissue by its photobiostimulatory effect. These effects occur as the tissue absorbs the laser light, which activates the intercellular cascade of signals, increasing cellular metabolism and anti-inflammatory properties [6].

The primary aim of this review is to comprehensively explore the mechanisms by which LLLT, also known as photobiomodulation (PBM), contributes to the acceleration of orthodontic tooth movement. This investigation seeks to elucidate the molecular and cellular processes influenced by LLLT, such as bone remodeling, inflammation modulation, and enhanced cellular activity, collectively facilitating more efficient orthodontic treatments. Additionally, this review aims to assess the clinical efficacy and safety of LLLT in orthodontics, offering a critical analysis of current literature to identify optimal treatment protocols and parameters. By integrating findings from various studies, this review endeavors to provide a foundational understanding for practitioners and researchers alike, aiming to bridge the gap between theoretical knowledge and practical application in orthodontic care. Ultimately, through evaluating the advantages and limitations of LLLT, this work seeks to offer insights into the future direction of accelerated orthodontic treatments, advocating for the development of standardized, evidence-based protocols to enhance patient outcomes in orthodontic practice.

## Review

Introduction to lasers

The word LASER is an acronym for light amplification by stimulated emission of radiation. The basic principle of laser is that an excited atom absorbs energy and releases photons that travel as coherent waves further emitting identical photons. This leads to the amplification and production of a laser beam [7].

There are usually two types of lasers used in dentistry. The first is the high-intensity laser, which is also known as a surgical laser (hard laser). Examples of surgical lasers are Co and Nd:YAG lasers. The second type of laser is LLL, such as diode and helium-neon lasers. Surgical lasers work on the principle of photothermal or ablative action on tissue, i.e., they cut the tissue by raising the temperature. In contrast, LLL is a cold light therapy, as it maintains a constant temperature and does not raise it above 36.5°C in the tissues throughout the procedure (soft lasers). This brings about non-thermal, biostimulatory effects on the bone and surrounding tissues. Other names for LLL include cold light therapy, PBM therapy, and laser phototherapy [8].

An LLL beam has three properties. The first and most important property is its monochromaticity, which means it emits light of only one particular color or wavelength. The second property is collimation, which means the light has a constant direction. The third property is coherency, which is described by the light waves moving in a similar manner. The laser beam is usually delivered in a non-contact manner. There are various modes of delivering LLL to the target tissues, including glass fiber optic cables, hollow tube-like waveguides, and handpieces (articulated arms). Additionally, different laser devices possess different emission modes of light, such as continuous wave mode, gated pulse mode, and free-running pulse mode. In continuous wave mode, the laser is emitted at a constant rate [9]. In gated pulse mode, there is an alteration of energy from time to time. Whereas, in the case of free-running pulse mode, the energy is released for a predetermined time followed by a duration when the laser is off [10].

When the tissues are exposed to LLL, four types of interactions occur between the target tissues and a laser beam. These are transmission, reflection, scattering, and absorption. In transmission, the laser beam passes through the tissue without causing any changes. Reflection occurs when a laser beam gets directed from the surface of the tissues without any effect. In scattering, the heat generated by lasers gets scattered to adjacent tissues leaving the target tissues unaffected. The last one is absorption, in which the target tissue absorbs the laser light of a particular wavelength and produces the desired effects. Once the tissues absorb lasers of a particular wavelength, it results in four types of biological effects. The first is the photothermal effect where the light energy is converted to heat energy. These types of effects are usually seen in surgical interventions. The second is the photochemical effect, which is produced when the lasers bring about a chemical reaction. The third effect is the photoacoustic effect, where a laser beam produces a shock wave causing an explosion of tissues. The last one is the photobiostimulating effect that changes the cellular atmosphere in tissues, thus enhancing remodeling, neovascularization, and vasodilatation [11]. The cold lasers used in orthodontics create photobiostimulating effects. These accelerate orthodontic tooth movement, reduce pain intensity, and encourage bone regeneration during expansion and distraction osteogenesis [12].

Mechanism of action of low-level laser therapy in accelerating orthodontic tooth movement

The molecular biological impact of LLL on orthodontic tooth movement is a vast topic. Various scientific studies have evaluated the diverse mechanisms by which LLLT affects cellular and tissue processes to produce accelerated orthodontic tooth movement. The literature supports the potential of LLLT to enhance bone remodeling, modulate inflammation, promote cellular activities, and influence gene expression, collectively facilitating faster and more efficient orthodontic treatments. The laser’s biostimulating effect is most noticeable during the proliferation phase of the cell [13].

The primary area of action of LLLT is mitochondria, mediated via cytochrome C oxidase (CCO), which is the terminal enzyme of the respiratory chain [14]. As the tissue is exposed to low-level lasers, CCO in mitochondria absorbs light. This excites wavelength-specific chromophores. These excited chromophores initiate signaling pathways that bring about three types of changes facilitating orthodontic tooth movement [15]. The first is by involving reactive oxygen species produced by mitochondria, which lead to bone and tissue remodeling [16]. Second, CCO directly increases the production of adenosine triphosphate (ATP). The availability of ATP is also brought about by inhibitory nitric oxide, a free radical considered an important signaling molecule in ATP production. This nitric oxide is responsible for angiogenesis and vasodilatation, thus increasing the velocity of tooth movement and providing analgesic effects [17].

Bone remodeling, driven by forces that promote bone absorption and deposition, is pivotal in orthodontic treatments. Osteoclasts and osteoblasts play critical roles in this process by facilitating bone resorption and formation, respectively. Several studies have demonstrated the effects of LLLT on stimulating bone remodeling. Observations in animal models suggest that the use of laser irradiation accelerates orthodontic movement by potentially increasing the number or activity of osteoclasts in the treated area [18]. Other studies have also highlighted the correlation between increased osteoclasts and osteoblasts, which could influence each other’s activities and contribute to bone healing and vascularization [17,18]. Interleukin-1 beta (IL-1β), which is released by fibroblasts, macrophages, cementoblasts, osteoblasts, lymphocytes, and osteoclasts, plays a critical role in bone metabolism [19]. This cytokine is notably prominent in the periodontal setting during the initial phase of orthodontic tooth movement, secreted primarily by osteoclasts in response to mechanical stress. Later in the orthodontic process, macrophages also contribute to its secretion. The accumulation of IL-1β has been observed in the compressed areas of the periodontal ligament. Its impact on osteoclast fusion, survival, and activation directly influences the efficiency of alveolar bone remodeling, thereby influencing the amount of tooth movement [20]. Additionally, IL-1β is closely associated with bone resorption as it stimulates the expression of receptor activator of nuclear factor κ B ligand (RANKL) in osteoblasts and periodontal ligament cells, encouraging the differentiation of osteoclast precursors [21]. A study conducted by Üretürk et al. [21] revealed that the laser-treated group exhibited higher levels of IL-1β compared to the control group. This study also established a positive correlation between IL-1β levels and the extent of tooth movement throughout the observation period [22]. Recent research suggests that LLLT may accelerate tooth movement by activating the molecular pathway responsible for osteoclast formation and essential in bone remodeling. This approach enhances the differentiation of cells that resorb bone, facilitating the necessary changes in bone structure to allow for quicker tooth adjustment [23].

Gonçalves et al. [24], alongside the study by Zhong et al. [25], extensively explored the potential of PBM in enhancing orthodontic treatments. Gonçalves et al. [24] focused on identifying the most effective PBM parameters, i.e., wavelengths (655, 810, and 940 nm), power densities (5 and 10 mW/cm^2^), and treatment schedules, to modulate key bone remodeling mediators such as alkaline phosphatase, osteoprotegerin, and RANKL. Their findings highlighted that specific PBM settings could significantly influence bone formation or resorption, offering crucial insights for orthodontic treatment optimization through targeted bone turnover stimulation. Complementarily, Zhong et al. [25] demonstrated that PBM significantly boosts orthodontic tooth adjustment, bone creation, and blood vessel growth through co-culturing MC3T3-E1 (osteoblast precursor cells) and HUVEC (human vascular endothelial cells), applying PBM to these co-cultured cells and rats undergoing orthodontic tooth movement. Both studies underscore the utility of PBM as an effective supplementary therapy in orthodontics, capable of accelerating tooth movement and enhancing bone health post-treatment through stimulation of bone and vascular development. Together, these investigations suggest the potential of tailored PBM protocols in revolutionizing orthodontic treatments, advocating for further research through animal model studies to unlock its full therapeutic potential.

The low-level laser therapy procedure in accelerated orthodontics

PBM is a completely painless procedure that does not require local anesthesia. Hence, it is very comfortable for the patient. Furthermore, the operator only requires basic training to be able to handle the device properly. Before commencing LLLT, an orthodontist evaluates the patient’s orthodontic needs, including the type of malocclusion, tooth movement required, and treatment goals [26]. A customized treatment plan that considers the inclusion of LLLT is then formulated. The specific protocol varies depending on the type of laser device, wavelength, power settings, and duration of exposure. Typically, LLL is applied to the targeted areas, such as the buccal and palatal area surrounding the teeth undergoing orthodontic movement [27].

The PBM device used for orthodontic tooth movement is a multipaneled system that emits cold polychromatic light of wavelengths between 450 nm to 850 nm. The device consists of a silicon mouthpiece, an LED array, an accelerometer, and a rechargeable battery. The silicon mouthpiece is made up of medical grade, soft, and waterproof silicon and needs to be placed between both arches. This mouthpiece is available in a universal size that fits all patients. The LED array, which is attached to this mouthpiece, emits infrared light of up to 850 nm wavelength. The device is provided with an accelerometer, which monitors the treatment time for the session, i.e., the duration for which the tissue will be exposed to the infrared beam. Once the exposure time is over, the light indicates that the treatment is done. The device is then removed from the oral cavity. The removable battery needs to be recharged from time to time.

The procedure should be performed at regular intervals depending on the severity of malocclusion. Throughout the treatment, the orthodontist closely monitors the progress of tooth movement and assesses the response to LLLT. It is important to note that while LLLT shows promise in accelerating orthodontic tooth movement, its efficacy can vary among individuals. The specific protocols and parameters for LLLT application might also differ based on the type of laser device used, patient responsiveness, and the orthodontist’s clinical judgment. However, there is currently no consensus on a predefined protocol for applying LLL to accelerate orthodontic tooth movement [28].

Efficacy of low-level laser in accelerated orthodontics

LLLT has a stimulating effect on orthodontic tooth movement. This effect includes increased neovascularization, collagen fiber synthesis, initiation and differentiation of bone remodeling, and an increase in ATP levels, thereby bringing about faster orthodontic tooth movement. Several studies have investigated the efficacy and effects of LLLT in accelerated orthodontics, shedding light on its potential benefits. Several studies have evaluated the impact of LLLT on accelerated orthodontics, and most of these have concluded that LLLT causes an increase in orthodontic tooth movement. Additionally, most studies have considered the split-mouth technique for LLL, which, in a way, rules out any patient-dependent factors that could affect the outcome of the therapy [28,29].

The first study that evaluated the effects of LLL on orthodontic tooth movement was conducted by Cruz et al. [30] in 2004. This study utilized the split-mouth technique in patients undergoing orthodontic treatment. One side of the patient’s maxillary arch received only mechanical activation for canine retraction. Conversely, mechanical activation was combined with irradiation with a diode laser light at the wavelength of 780 nm for 10 seconds at 20 mW with an energy of 53/cm^2^. This irradiation was done on days 0, 3, 7, and 14, each month for two months. The study discovered that tooth movement on the irradiated side was accelerated by 34% compared to the side without irradiation [30].

A similar study was conducted by Sousa et al. [31] using a diode laser at a wavelength of 780 nm at 20 mW with an energy of 231 cm^2^ for 10 seconds. This PBM was done for three days in three months. The velocity of tooth movement was almost 1.49 mm more on the irradiated side compared to the non-irradiated side [31].

In a 2013 study [32], involving 20 participants, including 14 girls and six boys, researchers investigated the impact of LLLT on the speed of tooth movement during orthodontic treatment. This treatment involved the removal of the upper first premolars, followed by the individual retraction of canines, and then collective retraction of the central and lateral incisors. Employing a split-mouth design, the study compared the effects of LLLT on one side of the mouth to a control group on the other side. The experimental side received LLLT with a 20 mW power and a dosage of 0.17 J/cm² on specific days (0, 3, 7, 14, 21, and 28), amounting to 10 total doses, with half applied from the buccal side and half from the palatal side. Gingival crevicular fluid was collected during each visit to assess nitric oxide levels. Results indicated an acceleration of orthodontic tooth movement on the LLLT-treated side compared to the control side, although nitric oxide levels in the gingival fluid remained largely unchanged [32].

Ekizer et al. [33] conducted a randomized controlled trial on 20 patients who required the extraction of first premolars followed by canine retraction using mini-implants to investigate the efficacy of LLLT in orthodontic treatments. The patients underwent LLLT for 21 consecutive days, receiving 20 minutes of treatment daily on the test side of their mouth. This method was directly compared with the control side, which did not receive any form of laser irradiation. The primary objective was to evaluate the speed of canine retraction and assess the stability of the mini-implants used in the procedure. According to the findings reported in the second and third months post-treatment, there was a significant enhancement in the rate of tooth movement and the stability of the mini-implants on the side subjected to LLLT, highlighting the potential benefits of incorporating LLLT into orthodontic treatment regimens [33].

In 2023, Özsoy et al. [34] investigated the effects of LLLT on tooth mobility during maxillary molar distalization in orthodontic treatment within a 12-week period. The research targeted 16 specific points on both the first and second molars, subjecting them to 10-second intervals of laser therapy at each point. A comparison with the contralateral control group revealed significantly greater tooth movement on the side treated with the laser. In contrast to the untreated side, the molars that underwent laser treatment exhibited 1.22 times more growth over 12 weeks [34].

Conversely, some studies have documented no significant difference in tooth movement between the LLLT and control groups without irradiation. In 2014, Kansal et al. [35] conducted a comparative clinical trial and found no statistically significant differences in the rate of tooth movement during canine retraction between the laser-treated and the control groups. The limited sample size of 10 patients in this study could have contributed to this outcome, possibly affected by variations in biological factors, bone characteristics, and the positioning of canine roots in the cortical plates. These variables could be better addressed with a larger sample size [35]. In a single-blind comparative clinical trial by Heravi et al. [36] LLLT was found to have no significant impact on the velocity of canine movement. The disparities in radiation parameters might be accountable for these findings [36]. Similarly, in 2015, Dalaie et al. [37] conducted a double-blind randomized comparative clinical trial, revealing that the effect of laser irradiation on the extent of tooth movement was not significant (p = 0.45). This result might be attributed to differences in radiation parameters. Additionally, the study’s small sample size of only 12 patients could have influenced its outcome [37].

These studies, among others, contribute to the ongoing discussion regarding the efficacy of LLLT in accelerated orthodontics. While some studies have not found substantial evidence supporting its use in expediting tooth movement or reducing discomfort, further research with standardized protocols and larger sample sizes may provide more conclusive insights into its effectiveness in accelerated orthodontics.

Safety of low-level laser in accelerated orthodontics

Scientific research consistently demonstrates the absence of adverse effects related to LLLT. A study by Youssef et al. [38] highlighted the safety of LLLT in pain management during orthodontic procedures. This study involved 15 individuals aged 14 to 23 who needed premolar extractions and canine retraction, comparing pain levels between sides of the jaw treated with LLLT to untreated sides. Results indicated a significant reduction in pain on the LLLT-treated sides, emphasizing the safety and efficacy of the method in minimizing orthodontic treatment discomfort [38]. Additionally, another investigation found no radiographic signs of damage to dental or periodontal tissues from LLLT use [39].

In related research, Rossi et al. [40] explored the impact of PBM on root resorption in orthodontic patients using clear aligners, with no notable difference in root resorption observed between the group using PBM and the control group. This outcome suggests that PBM’s adjunctive application in orthodontic treatments using clear aligners does not escalate the risk of orthodontically induced external root resorption; however, caution is advised when interpreting these results due to the limited sample size and possible measurement inaccuracies [40]. Olek et al. [41] also reported that photodynamic therapy could decrease microbial levels in patients with fixed orthodontic appliances, presenting a viable alternative to traditional periodontal maintenance practices [41].

Advantages of photobiomodulation in orthodontic treatment

PBM offers a range of benefits that position it as a superior option to traditional orthodontic treatments, particularly in terms of patient comfort and safety. The non-invasive nature of PBM is one of its most notable advantages, eliminating the need for surgical interventions, thereby reducing the risk of complications [39]. This aspect alone makes PBM a safer choice, especially when compared to methods that involve electrical stimulation in moist environments [38-41]. Additionally, PBM’s precision in action and control, facilitated by the use of specific wavelengths tailored to individual treatment plans, enhances its safety profile and effectiveness [38-41]. Unlike traditional orthodontic methods, PBM does not result in tissue damage, infection, blood loss, or discomfort, providing relief from these conditions instead [38-41]. Furthermore, its capacity to promote tissue healing, angiogenesis, bone remodeling, and accelerated tooth movement underscores its value as an adjunctive therapy in orthodontics [24,25].

Limitations of photobiomodulation in orthodontic treatment

Despite the advantages, the application of PBM in orthodontics is met with several challenges. The absence of standardized treatment protocols is a primary limitation, introducing variability in treatment parameters and outcomes [28,42-46]. This variability is compounded by individual differences in response to PBM, which can hinder the development of universal treatment guidelines [28]. The need for multiple sessions to achieve noticeable results may also deter patients seeking quicker solutions. Moreover, the effective utilization of PBM devices requires specific clinician training to prevent potential misuse or excessive use, which could lead to tissue damage. Another significant barrier to the widespread adoption of PBM is the cost of laser equipment, which, along with the need for intensive safety training, may limit accessibility for some practices and patients [24,25,38-46].

Future scope

The future scope of LLL applications in accelerated orthodontics is promising, with ongoing research and advancements suggesting several potential developments. Advancements in laser technology may lead to more precise and targeted applications of LLLT. This could enable orthodontists to precisely control the energy levels and wavelengths for optimized tooth movement, thus enhancing treatment efficiency and minimizing side effects. In addition, tailoring LLLT protocols based on individual patient characteristics, such as bone density, tissue response, and tooth movement requirements, should become standard practice because personalized treatment plans would lead to more predictable and efficient outcomes. Furthermore, the integration of LLLT with other emerging technologies, such as photobiomodulation, nanotechnology, or regenerative medicine, might offer synergistic effects. From a patient’s point of view, continued advancements in LLLT might pave the way for non-invasive or minimally invasive orthodontic treatments. This could appeal to patients seeking less invasive alternatives to traditional braces or aligners while achieving accelerated tooth movement. Further research incorporating extensive clinical trials and focussing on standardizing LLLT protocols would solidify its efficacy and safety in accelerated orthodontics. This validation would encourage wider adoption among orthodontic practitioners. Apart from these benefits, a collaboration between orthodontists, laser scientists, tissue engineers, and biomaterial experts could foster innovative approaches. This collaborative effort could result in novel applications and materials designed specifically for enhancing the effectiveness of LLLT in orthodontics. As research continues to explore the potential of LLLT in accelerated orthodontics, these developments could transform treatment approaches to offer quicker, more comfortable, and more personalized orthodontic solutions for patients in the future.

## Conclusions

In the modern pursuit of minimally invasive and comfortable orthodontic treatments, LLLT stands out for its non-invasive nature and potential to accelerate tooth movement, offering patients quicker results with reduced discomfort. Notably, LLLT also diminishes pain perception, adding to its appeal. However, the technique’s drawbacks include the high cost of laser equipment and the need for specialized training to ensure safe usage. While current research broadly supports the efficacy of LLLT in orthodontics, a lack of consensus on standardized application protocols persists due to varied treatment parameters. To further validate the benefits of LLLT, more extensive randomized controlled trials are essential. Such studies could help establish standardized guidelines, making LLLT a more universally accepted and effective treatment option in orthodontic care.
